# Four-level evaluation of health promotion intervention for preventing early childhood caries: a randomized controlled trial

**DOI:** 10.1186/s12889-017-4783-9

**Published:** 2017-10-02

**Authors:** Leila Basir, Bita Rasteh, Ali Montazeri, Marzieh Araban

**Affiliations:** 1 Department of Pediatric Dentistry, School of dentistry, Ahvaz Jundishapur University of Medical Sciences, Ahvaz, Iran; 2 School of dentistry, Ahvaz Jundishapur University of Medical Sciences, Ahvaz, Iran; 3 Mental Health Research Group, Health Metrics Research Center, Iranian Institute for Health Sciences Research, ACECR, Tehran, Iran; 4 Social Determinants of Health Research center, Ahvaz Jundishapur University of Medical Sciences, Ahvaz, Iran; 5 Department of Health Education and Promotion, Public Health School, Ahvaz Jundishapur University of Medical Sciences, Ahvaz, Iran

**Keywords:** Perceived threat, Health literacy, Oral health, Early childhood caries, Health education, Health promotion

## Abstract

**Background:**

Early childhood caries (ECC) is the most common dental disease among children, which can affect children’s primary teeth during their teething. This study evaluates an intervention for preventing early childhood caries in a pediatric population in Ahvaz, Iran.

**Method:**

The population of this study (IRCT2017070210804N10) consists of 104 women with 12 to 36 months of age without dental caries referred to a health care center in Ahvaz, Iran. The children were randomly assigned to either an experimental or control group in equal numbers. First, the demographic information of participants was collected through a questionnaire containing components of perceived threat, health literacy, and oral health behaviors using a valid and reliable questionnaire. The ECC status of the children was established by a dentist. Control group received “standard well baby care”. The experimental group received standard well baby care in addition to educational interventions, including lecture and group discussion. After 6 months, the participant completed the questionnaire for the second time, and the children’s teeth were reexamined. Data were analyzed using SPSS version 15 at a significance level of *p* < 0.05.

**Results:**

The mean ages of women and children were 31 ± 6.68 years and 18 ± 7.21 months, respectively. Before the intervention, no significant difference was documented between the groups for the study variables, *p* > 0.05. However, after the intervention, a significant difference was observed between the perceived threats (41.15 ± 4.46 in the experimental group and 38.26 ± 4.21 in the control group, *p* = 0.001), health literacy (20.98 ± 2.15 in the experimental group and 19.76 ± 2.70 in the control group, *p* = 0.01), oral health behaviors (7.75 ± 2.30 in the experimental group and 6.15 ± 2.65 in the control group, *p* = 0.01), and the incidence of ECC (13% in the experimental group and 35% in the control group**,** *p* = 0.001).

**Conclusion:**

This intervention had positive effects on the perceived threat, health literacy, and health behaviors; and the intervention could reduce the incidence of ECC. The finding of this study provided a suggestion for evidence-based decision-making processes regarding ECCs prevention programs.

**Trial registration:**

IRCT2017070210804N10 (retrospectively registered)

## Background

Early childhood caries (ECC) is the most common childhood disease among children [1–3], which can affect their primary teeth upon their teething [4]. The American Academy of Pediatric Dentistry (AAPD) defines ECC as the presence of one or more decayed (non-cavitated or cavitated), missing (as a result of caries), or filled tooth surfaces in any primary tooth in a child 71 months of age or younger. AAPD also specifies that, in children younger than 3 years of age, any sign of smooth-surface caries is indicative of the severe early childhood caries (S-ECC) [5]. A comprehensive review, including studies from Europe, Africa, Asia, the Middle East, and North America, showed that the prevalence of ECCs in socioeconomically-deprived groups could be as high as 70% [6]. In an article by Tootooni et al. (2015) the prevalence of ECC among children between 2 and 3 years old was reported to be very high, with 61.1% of samples displaying pitted caries [7]. Despite the improvement in dentistry practice, ECC still remains as remains a serious challenge for health care providers [8].

Early caries in primary teeth causes many difficulties, including problems with chewing and speaking, pain, psychological problems, and negative effects on the child’s growth, weight, and quality of life both within the family and in society [9]. ECC is preventable with appropriate educational interventions and health promotion [10]. In a study conducted by Manchanda et al. at Oxford University, providing mothers with oral health education led to a reduction in dental caries in children [11].

Nutbeam (1998) believed that health promotion interventions are conducted on four levels. The first level includes health promotion activities, mainly consisting of health education. The second level includes discussing the health promotion outcomes and health literacy. The third level includes intermediate health outcomes or behavior. Finally, the fourth level involves examining health indicators, such as the prevalence of caries or dental plaque scores. Education provides opportunities for learning aimed at improving health literacy, and subsequent ability of individuals to improve their health behaviors. Doing so, individuals’ quality of life and health indicators will progress. It should be noted that there is not any linear relationship among these processes [12].

Conducting theory-based interventions is among the preconditions for ensuring the effectiveness of an intervention. In this regard, the focus has recently moved from the individual behaviors toward multi-level behaviors [13]. Health promotion consists of two main parts: a change in environment or some regional policies, and health education [14]. Health education is still considered as a basic component of health promotion and is widely recognized as a tool for changing health behaviors [15]. Previous studies have shown that people’s behaviors are affected by factors such as a perceived susceptibility, diagnosis and estimation of each person’s susceptibility to a disease and exposure to risk and perceived severity, and feelings about the seriousness of getting a disease or not treating it. More precisely, a perceived threat was more effective in the acquisition of oral health behaviors [16]; therefore, considering these theoretical structures can be important in oral care education. Education should be conducted in a way that individuals acquire knowledge, a positive attitude toward the particular health issue, health literacy, and the necessary skills to carry out the elements of the health program [12]. These requirements ensure that people can process and understand basic health information properly and make correct decisions regarding their health; these behaviors are known as health literacy [17, 18]. These desirable beliefs and decisions should lead to the adoption of healthy behaviors and should ultimately improve oral health-related indicators [15]. Despite rigorous treatment and examination in pediatric dentistry practice, little attention has been paid to oral health education. Given the suggested deficiency in oral health, on one hand, and to fill this gap in pediatric dentistry, on the other, conducting a health promotion intervention seems to be necessary.

Education is frequently the missing element in the care provided to a population. To accomplish the goal of educating a population about dental health care, we set up a final-year dentistry student to provide dental health education to women with children aged 12–36 months old. Based on the previously defined issues, the most significant questions arose: Could health promotion intervention improve children’s oral health and could oral health education consequently reduce ECC? Therefore, this study evaluated the effectiveness of a health promotion intervention aimed at ECC prevention in the west of Ahvaz (southwest Iran).

## Methods

This experimental study was carried out through a parallel-group design in the health care clinic of western Ahvaz, located in the southwest of Iran, from 25 April 2015 to 10 April 2016.

### Participants

#### Eligibility criteria for participants

The study population included women with children aged 12–36 months old without caries (caries-free children were selected to reduce any biases in relation to considering fluorosis or enamel hypoplasia as ECC) and 104 women with children with an age of 1 to 3 years old. The participants were randomly placed in either the experimental group (*n* = 52) or the control group (*n* = 52) on a 1:1 basis. Inclusion criteria were as follows: proficiency in the Persian language, willingness to participate in the study, and having no history of disease in the mothers or the children (due to their effect on dental health, such as epilepsy, cancer, diabetes, use of anticholinergic drugs) according to health care records. Children must have at least 8 completely erupted teeth, 4 maxillary anteriors, and 4 mandibular anteriors. Women were excluded if they: 1. do not attend the educational session, 2. move to another city, and 3. got diagnosed with a health condition that interferes with education.

#### Sample size

Sample size was estimated by a statistical power analysis. The primary outcome of this study was to measure score changes that might be observed for perceived threat [19]. Thus, to detect a 1.5-point increase in the baseline perceived threat score at 5% significance, the study would require a sample of 52 participants per each group (intervention and control groups); thus, the study would have a power of 90%.

#### Study setting

The study was conducted in the maternal-child health wards of western Ahvaz, Iran. This center is a large comprehensive health that gives health services to a large number of people. The participants were recruited through local advertising.

#### Intervention

Based on information obtained from a pilot study performed by the authors, an educational intervention (consisting of one individual session and a group (4–6 person) lasting for half an hour) was designed with the aid of women referring to the health center for monitoring their children’s growth. To save time, sessions were conducted when mothers were waiting for their children’s growth monitoring to be performed. Sessions were held based on question and answer, lectures, group discussions. Next, after in-person brief interventions, educational short message service (SMS) reminders were sent fortnightly for 6 months to the women to keep them motivated about their children’s dental care. During the educational intervention, the women were supplied with basic information about observance of children’s oral health (such as appropriate nutritional patterns, tips about how to breastfeed the child at night, and how to brush or clean children’s teeth). By providing statistics on caries and their complications, we drew the women’s attention to this issue. Next, the women were asked to evaluate whether prevention or treatment was considered as a better form of dental care. In addition, photos of children with either healthy or decayed tooth were showed to them and they were asked to evaluate, which child’s smile is more beautiful. The costs of preventive behaviors versus dental treatments were explained in simple examples for the women and they were asked to evaluate which one is better: taking preventative care or treatment.

The educational intervention was based on reliable materials and sources from the Ministry of Health, while prepared slides were confirmed by an expert in the area of educational technology. In addition, participants were given a pamphlet containing brief and important tips on the promotion of educational items, and the need for oral health care for their children. The cost of educational materials was $2 per person. Before the educational session, information about both groups was gathered, and education was delivered to the experimental group in addition to standard childcare procedures. The control group received standard care “well baby program”, which includes the Expanded Program on Immunization (EPI) [20], child growth and development. Through this program, a health care provider measures child’s height and weight and marks them on a growth chart. The health care provider uses the chart to determine how the baby is growing compared with other children of the same age and gender. Also, she/he asks mothers questions about how her child is doing - whether she’s hit typical milestones, is active, and is feeding and sleeping okay to screen some behavioral or intellectual problems. Well-baby visits are a chance for mothers to address their concerns.

After 6 months, follow-up information was gathered from both the intervention and control groups using a questionnaire. Then, the children’s teeth were examined by a dentist to check for ECC. We considered tracking time as 6 months, as a period of 3–6 months is considered desirable in behavioral science studies [15, 21]. Besides, this duration could affect oral health status [22].

### Measures

#### Demographic data sheet

The demographic data sheet included mother’s age, the child’s age, the child’s gender, and the mother’s and husband’s occupations and education. Participants were also asked whether they had health insurance and to disclose their perception of family’s monthly incomes. The economic status was measured on a four point Likert type scale consisting of poor, fair, good, and excellent scores [23].

#### Questionnaire: Perceived threat

This section in the questionnaire consisted of 10 statements derived from relevant literature [15, 19, 24, 25] that measure the perceived susceptibility and severity of the effects of ECC. The score of each question was calculated from 1 to 5, using a five-point Likert scale ranging from “I strongly agree” to “I strongly disagree.” The scores ranged from 10 to 50, and earning a higher score indicated a higher perceived threat. For example, the statement, “a child must be taken to a dentist, only when his tooth hurts him,” was one of the questionnaire’s statements.

#### Questionnaire: Oral health literacy

This section consisted of 5 statements that appraise oral care health literacy derived from relevant literature [26, 27]. Each question was scored within a range of 1–5, using the same five-point Likert scale ranging from “I strongly agree” to “strongly disagree.” The scores ranged from 5 to 25, and earning a higher score indicated higher oral health literacy. For example, the statement, “since the child will lose his/her primary teeth, so there is no need to take care of them” was one of these statements.

#### Questionnaire: Oral health behaviors

The health performance of women regarding their babies’ teeth included three questions derived from [11, 14, 19]:

- “Do you take your child to the dentist for periodic examinations?”
- “Do you clean the child’s teeth after eating sugary foods?”
- “How many times do you brush your child’s teeth or clean them each day?”

Questions were answered using a five-point Likert scale, which included “always,” “often,” “sometimes,” “rarely,” and “never” for the first and second questions and the third, was ranked from 1 to 4 and was answered as follow as: “never”, “once a day”, “twice a day”, “three times a day”. The scores ranged from 3 to 14. Again, earning a higher score suggest more desirable conditions.

### Measurement of ECC

Caries was diagnosed visually, after drying and cleaning the teeth with sterile gauze. Dental examination was conducted using the natural light, a mirror, and a probe in a knee-to-knee position. ECC was diagnosed based on WHO criteria [28]. ECC in this study was considered as the presence of dental caries in any surface of at least one tooth in the primary dentition (including 8 maxillary or mandibular anterior incisors) in children 12 to 36 months old. We represented only white spot including non-cavitated lesions categorized as D1 = initial caries/caries limited in enamel; the lesion demonstrates whitish/yellowish opaque with/without micro-cavity but no softened floor wall [29]. Examinations were performed by a dentist blinded to group assignment. A dentist assessed the presence of ECC of a child twice and recorded the findings on a checklist. These examinations conducted on ten 12–36-month-old children excluded from study samples. There was a 1-day interval between examinations. The intra-examiner reliability as measured by Kappa coefficient was found to be satisfactory (κ = 0.8).

### Reliability and validity of the questionnaire

The validity of the questionnaire was confirmed by 15 experts in the fields of pediatric dentistry, midwifery, public health, and health education while its reliability was measured using Cronbach’s alpha coefficient.

To ensure comprehension, the questionnaire wording and comprehension were evaluated by the participants; the questionnaire was completed by 10 women, and the questionable items were corrected. For example, the item “I do believe that there is no need to refer to a dentist during childhood” was replaced by “I do believe that there is no need to refer to a dentist during the early childhood.” The experts were asked to comment on the necessity and relevance of the items regarding oral health care for children to estimate the content validity ratio (CVR) and the content validity index (CVI), respectively. The necessity of an item was judged using a three-point rating scale as follows: not essential, useful but not essential, and essential. Following the experts’ assessments, the CVR total scale was calculated. The CVI was estimated by experts’ ratings of the items’ relevancy, simplicity, and clarity on a 4-point Likert scale. According to Lawshe (1975), the CVR for the scale equal to or greater than 0.59 was considered satisfactory [30]. The CVI for each item was calculated, and values equal to or greater than 0.80 were considered acceptable. The scales demonstrated the high content validity and reliability with a CVR = 1. The internal consistency of the questionnaire was investigated using Cronbach’s alpha, and the reliability of the questionnaire was 087, 080, and 0.70 for perceived threat, health literacy, and behavior, respectively.

#### Randomization

Randomization was achieved using opaque sequentially numbered envelopes developed from a random number generator. A research assistant who was not involved in the recruitment of participants prepared the envelopes. The allocation of eligible participants was performed by a health practitioner who was not a member of the research team at the health center. BR, the research statistician, and pediatric dentist, and LB were remained blind throughout the study and analysis.

#### Procedure for recruitment and application of the intervention

Each day, and for an eight-month period, a dentistry student, BR, obtained a list of all women attending clinics for child growth monitoring, according to a scheduled daily program. Participants who did not meet the inclusion criteria were excluded from the study. Next, BR went through the clinical records of the remaining participants to assess their eligibility. At this stage, women with medical conditions affecting their probable oral health status were omitted. Participants eligible for participation were asked to provide informed consent and completed a questionnaire. After baseline measures were completed, BR introduced the participants to MA and left to ensure that she remained blind to the group allocation. A research nurse randomized participants into either the experimental or the control groups. BR presented the educational intervention to the experimental group in the waiting room. After 6 months, participants in both groups completed the questionnaires and were examined again, during the research follow-up.

### Ethics

This study is a secondary data analysis obtained from an earlier research, confirmed by the Ethics Committee of Ahvaz Jundishupur University of Medical Sciences. At the end of this study, to observe ethical principles, women in the control group were also given adequate education about their children’s oral health care.

### Statistical analysis

All data analyses were conducted according to a pre-established analysis plan through SPSS 15 (SPSS, Inc., Chicago, IL, USA). First, the normality of scores was evaluated and measured, using the Kolmogorov-Smirnov test and standard deviations (SDs). The proportions were compared using the chi-square analysis. The Spearman test was used to examine the correlation between demographic variables and health behaviors. Analytical tests, such as an independent t-test and a paired t-test, were used to compare these two groups. Results with *P* < 0.05 were considered statistically significant.

## Results

### The study sample

**Fig. 1:**
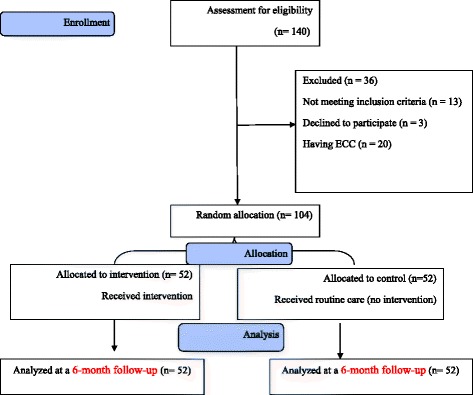
Flow diagram of the participants

In this work, 114 women were contacted, with 3 refusing to participate and 33 not meeting inclusion criteria. Not having enough time was the main reason for rejecting the program.; however, there was no statistically significant difference between demographic variables of those women and the participants (Fig. 1).

Mean and SDs for the women’s and the children’ ages were 31.6 ± 6.68 years and 18 ± 7.21 months, respectively; and there was no significant difference between the two groups (*p* > 0.05). A chi-square test was used to compare the employment statuses of women and fathers and their insurance status, where no significant difference was observed between the experimental and control groups (*p* > 0.05). In addition, 27% of women reported that they had never visited a dentist before. Table 1 shows other demographic characteristics of the control and experimental groups. The results of a Spearman test did not show a significant correlation between two groups for variables including mother’s age, child’s age, the status of insurance, or education, and oral health behaviors (*p* < 0.05).

**Table 1:** Characteristics of the studied participants of two groups at baseline

Group	All participants	Intervention (n = 52)		Control (n = 52)		
		Mean (SD)	Number (%)	Mean (SD)	Number (%)	*P* value
Women age (year)	31 ± 6.68	32.07 (7.56)		31.69 (6.59)		0.67*
Infant age (month)	18 ± 7.21	19.44 (8.83)		18.51 (8.79)		0.49*
Having had dental visit						0.85**
Yes	29 (27.88)		15 (28.84)		14 (26.92)	
No	75 (72.12)		37 (71.16)		38 (73.08)	
Child sex						0.99**
Male	52 (50)		26 (50)	26 (50)		
Female	52 (50)		26 (50)	26 (50)		
Education						0.74**
≤ High school	10 (9.6)		4(7.7)		6 (11.5)	
> High school	94 (90.4)		48 (93.3)		46 (88.5)	
Husband’s education						0.73**
≤ High school	10 (9.6)		4 (7.7)		6 (11.5)	
> High school	94 (90.4)		48 (93.3)		46 (88.5)	
Income						0.81**
Less than fair	12 (11.53)		5 (9.6)		6 (11.53)	
Fair and better than fair	92 (88.47)		47 (90.4)		46 (88.47)	

*Results obtained from t-test

**Results derived from chi-square

To compare the status of perceived threat, health literacy, and health behavior of women in both control and the experimental groups, an independent t-test was used to measure the data before and after the educational intervention. Before the intervention, there was no significant difference between the two groups in terms of perceived threat, health literacy, and oral health behaviors (*p* > 0.05); however, after the intervention, the difference was significant (*p* < 0.05). On the other hand, after the intervention, a significant difference was observed between perceived threats (41.15 ± 4.46 in the experimental group and (38.26 ± 4.21 in the control group, *p* = 0.001), health literacy (20.98 ± 2.15 in the experimental group and 19.76 ± 2.70 in the control group, *p* = 0.01), and oral health behaviors (7.75 ± 2.30 in the experimental group and 6.15 ± 2.65 in the control group, *p* = 0. 01). Paired t-test results for inter-group differences showed no statistical differences before and after the intervention for the two groups on other issues such as the concern for a perceived threat, health literacy, or oral health behaviors (*p* < 0.05). However, the comparison of decayed teeth between the two groups, after the intervention, revealed statistically significant results (*p* < 0.05) (Tables 2, 3, 4, 5).

**Table 2:** Comparisons of perceived threat between two groups over the study period

Groups Time	Intervention Mean(SD)	Control Mean(SD)	*p*-value*
Pre-Test	38.92 ± 5.32	37.88 ± 4.72	0.27*
6 months follow-up	41.15 ± 4.46	38.26 ± 4.21	0.001*
*P*-value**	0.002**	0.44**	–

*Results obtained from t test

**Results obtained paired t test

**Table 3:** Comparisons of oral health literacy two groups over the study period

Groups time	Intervention Mean (SD)	Control Mean (SD)	*p*-value*
Pre-Test	19.63 ± 2.27	19.73 ± 2.68	0.84*
6 months follow-up	20.98 ± 2.15	19.76 ± 2.70	0. 01*
*P*-value**	0.001**	0.91**	–

*Results obtained from t test

**Results obtained paired t test

**Table 4:** Comparisons of oral health behavior between two groups over the study period

Groups time	Intervention Mean(SD)	Control Mean(SD)	*p*-value*
Pre-Test	5.57 ± 2.53	5.59 ± 2.59	0.97*
6 months follow-up	7.75 ± 2.30	6.15 ± 2.65	0. 01*
*P*-value**	0.001**	0.06**	–

*Results obtained from t test

**Results obtained paired t test

**Table 5:** Comparisons of ECC incidence between two groups at the follow-up

Groups time	Intervention Number (%)	Control Number (%)	*p*-value*
ECC incidence at follow-up	7 (13)	17 (35)	0.001

*Chi-square test

## Discussion

This study is among few works investigating the effectiveness of health promotion interventions in preventing ECC at four recommended levels. The results of this study showed the effectiveness of the educational intervention on women’s perceived threat regarding ECC. In line with our study, the theory-based studies by Shamsi et al. [19] and Shhnazi et al. [25] proved the perceived threat (including perceived susceptibility and severity) of the need for oral care, specifically regarding dental care for women. However, a study by Moinie et al. [31] demonstrated that the educational program failed to improve perceived susceptibility scores in results that were not consistent with the results of this study. That difference may be explained by their use of peer education, specifically, child-to-child, which led to less effectiveness when compared to education by dentists; as a result, there was less susceptibility to perceiving a threat to oral health as compared to our study. Regarding the increased perceived threat for women with children younger than 3-years-old, family health personnel and dentists must act to increase the education of the women, by providing statistics on the prevalence of ECC in children younger than 36 months of age, their complications, and their effects on many aspects of general well-being.

The second level of intervention evaluated the effect of specific education on oral health literacy. No other study was found that evaluated the impact of education on oral health literacy; however, a study by Meppelink revealed a positive impact of educational intervention on general health literacy and health behavior [32]. In this study, simple educational explanations of materials in words, and in educational pamphlets, along with the chance to interact with the dentist, helped participants promote their oral health literacy. Previous studies have reported that using simple and understandable materials also helps improve health literacy [33, 34].

This study identified the improved performance of oral health behaviors for children. The study of Hajimiri et al. revealed that education based on a health belief model improves the performance of women who brush teeth of their 3 to 6-year-old children; these findings are consistent with the present study [24]. In addition, Manchanda et al. argued that oral health education for women with children between 6 to 18 months old improved the performance of women brushing for their children [11]. The results of a study by Huebner also showed that educational interventions increase brushing, which is consistent with the findings of this research. Although the study of Aljafari et al. highlighted that increasing information can improve oral health performance [14], Glanz et al. emphasized that mere information is not enough [15]. In addition to providing education, improving clients’ beliefs and skills were important in adopting healthy behaviors. In this study, participants took advantage of health promotion approaches, such as improvement of their skills in oral health behaviors, including learning how to brush their babies’ teeth.

Health promotion interventions should be effective in changing health-related consequences. In this study, less tooth caries was observed in the primary teeth of children within the educated participants in the experimental group. Clearly, oral health education given to women with children younger than 36 months of age can improve women’s performance in brushing and cleaning their children’s teeth, and reduce the incidence of early caries. In a study conducted by Medeiros et al., increasing information and motivation of women improved the oral health status of their children [35]. Reduction of ECC, as a result of the educational intervention, has been also reported in previous studies [36].

The strengths of this study were comprised of forming a primary care intervention that can be generalized to similar primary care settings; besides, the interventions included are feasible and inexpensive. Since dental schools need to respond to epidemiological challenges with enhanced education methods, the results of the current study might help dental schools overcome the challenge of increasing ECCs. Regarding the lack of correlation between health behaviors and demographic variables, we can utilize the contents of the present intervention for the population, regardless of their demographic characteristics.

### Limitations

Not measuring the impact of the intervention on reading and calculating other dimensions of health literacy are two main limitations of the present study. Considering that literacy is closely related to the level of education of individuals, this limitation cannot much affect the results. Another limitation was that we did not assess the babies’ feeding methods. However, since more than 80% of women in Ahvaz apply a mix of breastfeeding and bottle feeding [37], this limitation might not affect the validity of the study very much. Another limitation of this work is not performing dental surfaces examination. Finally, the results of the current study might not be generalized to all pre-school children since we entered caries-free children.

### Direction for future research

Further studies among all pre-school children including children with dental caries considering the above-mentioned limitations are recommended to confirm the results of the current study. Besides, applying a theory-based electronic health education to improve oral health should be considered for future studies.

## Conclusion

The four-level evaluation of health promotion conducted in this work had a desired impact on the perceived threat, health literacy, and oral health behaviors and reduced the incidence of ECCs in children. Because many health problems have similar risk factors, promotion of oral health can improve the status of overall health and the quality of life for many individuals. The finding of this study provided a suggestion for evidence-based decision-making processes regarding ECCs prevention programs for future preventive and social dentistry interventions. Furthermore, it is suggested integrating children oral healthcare with well-baby care program.

## Abbreviations

CVI: Content Validity Index
CVR: Content Validity Ratio
ECC: Early Childhood Caries
